# Focal Laser Photocoagulation in Non-Center Involved Diabetic Macular Edema

**Published:** 2014

**Authors:** Irfan Perente, Zeynep Alkin, Abdullah Ozkaya, Doukas Dardabounis, Tulin Aras Ogreden, Aristeidis Konstantinidis, Konstantinos Kyratzoglou, Ahmet Taylan Yazici

**Affiliations:** 1 Beyoglu Eye Training and Research Hospital, Bereketzade Cami Sok., Istanbul, Turkey,; 2 University Hospital of Alexandroupolis, Greece,; 3 Suleymaniye Women Health Hospital, Istanbul, Turkey

**Keywords:** Diabetic Macular Edema, Laser Photocoagulation, Focal Macular Laser Photocoagulation, Visual Acuity

## Abstract

This study was performed to evaluate the functional and anatomic outcomes of focal macular laser photocoagulation in eyes with non-center involved macular edema (non-CI ME). Forty-nine eyes of 43 patients with non-CI ME were included. Focal macular laser photocoagulation was conducted on twenty-nine eyes of 25 patients, while 20 eyes of 18 patients with non-CI ME were followed without treatment and served as the control group. Data relating to best corrected visual acuity (BCVA; Early Treatment Diabetic Retinopathy Study) and central subfield thickness (CST), inner zone thickness (IZT), outer zone thickness (OZT), and total macular volume (TMV) as determined by optical coherence tomography (OCT) were collected and compared between the groups. At 12 months, VA decreased by a mean of 0.4 letters in the treatment group and 3.3 letters in the control group (p=0.03). Gain in VA ≥5 letters was noted in 6 (21%) of the eyes in the treatment group versus 1 (5%) eye in the control group (*p*=0.12). At 12 months, average IZT decreased by 22.6 microns in the treatment group and increased by 10.9 microns in the control group (*p*<0.001). The treatment group revealed significant reduction in CST, average OZT, and TMV as compared to the control group at 12 months (all *p*<0.05).Generally, focal laser photocoagulation may have more favourable visual outcomes in this specific group of diabetic patients than does observation. In addition, focal laser treatment provided better outcomes with improvement in OCT parameters as compared to the control group.

## INTRODUCTION

Macular edema (ME) is the most common cause of visual impairment in diabetic patients (1). The Early-Treatment Diabetic Retinopathy Study (ETDRS) revealed that focal/grid laser photocoagulation reduced the risk of moderate visual acuity (VA) loss by approximately 50% in eyes with clinically significant ME that involved or threatened the center of the macula 3 years following the treatment (2). Given the importance of macular edema as a leading cause of visual impairment in the diabetic population, there is still a need for further investigation to evaluate early treatment options. In an attempt to explain the discrepancy between VA and predictive factors for outcomes of laser treatment, central macular thickness only explains 27% of the variability in VA. Although central macular thickness was found to be the most predictive factor, additional factors like angiographic leakage at the inner subfields also contribute to the loss of VA (3). Recent treatment recommendations for diabetic macular edema (DME) are based on involvement of the center of the macula (4). According to the current ETDRS guideline, focal/grid laser photocoagulation remains the recommended first-line therapy for DME without center involvement (5).

 Retinal thickness has traditionally been assessed by ophthalmoscopy and slit lamp biomicroscopy with contact or noncontact lenses, as well as stereoscopic fundus photographs (6). Recently, optical coherence tomography (OCT) has achieved importance for detection of subtle changes associated with the disease process and for providing quantitative estimation of retinal thickness within each of the nine subdivisions of the macular area (7). In clinical practice, decisions on treatment continuation, interruption, and re-initiation are mostly based on the combination of OCT and VA. In recent studies, the extent of agreement between OCT and fundus photographs has suggested that macular photocoagulation can be guided by the retinal thickness map from OCT (8). 

 In this retrospective study, we compared the changes in VA, macular thickness and volume parameters measured with OCT in patients with non-center involved macular edema (non-CI ME) treated with focal laser photocoagulation with patients who received no treatment. 

## METHODS

The data were collected from medical records of patients admitted with non-CI ME to the Retina Clinic of Beyoglu Eye Training and Research Hospital from June 2010 to August 2011. All patients had a follow-up time of at least 12 months. The treatment group comprised the eyes that had undergone focal laser photocoagulation and the control group comprised the eyes that were followed without treatment. Written informed consent explaining the potential risks and benefits of the procedure was obtained from the patients in the treatment group. 

 Patients older than 18 years, with non-proliferative diabetic retinopathy secondary to type II diabetes mellitus were included in this study. The eligibility criteria were as follows: (1) best corrected visual acuity (BCVA) score ≥19 letters (20/400 or better), (2) metabolic control of hyperglycemia being demonstrated by the level of glycosylated hemoglobin (HbA1c) ≤8.0%, (3) previously untreated DME characterized by a central subfield thickness (CST) ≤250 microns and ≥300 microns in at least one of the four inner subfields on the fast macular map scan, (3) well-defined focal areas of leakage from microaneurysms located between 500 and 3000 microns from the center of the macula revealed by fluorescein angiography (FA) and increased fluorescein leakage from microaneurysms positively correlated with increased retinal thickness on OCT without evidence of macular ischemia. 

 Eyes were not included if they had vitreoretinal interface abnormalities, an enlarged foveal avascular zone on FA, other macular pathologies such as age-related macular degeneration, retinal vascular occlusive disease, or major ocular surgery such as cataract extraction and vitrectomy within the last 6 months. 

 Each patient underwent a complete ophthalmic examination including best corrected visual acuity (BCVA) measurement with the ETDRS chart, intraocular pressure measurement using applanation tonometry, slit-lamp biomicroscopy, dilated fundus examination, fundus photography, FA via Heidelberg Retinal Angiograph (Heidelberg Retina Angiography; Heidelberg Engineering, Heidelberg, Germany), and OCT imaging (Stratus OCT 3000, Carl Zeiss, Meditec Inc., Dublin, CA, USA) at baseline. Best corrected visual acuity measurement and OCT imaging were repeated in all study eyes at the 3, 6, and 12-month follow-up visits.

 All eyes in the treatment group had undergone focal laser photocoagulation with 532 nm argon laser (SL-130; Zeiss-Humphrey systems, Carl Zeiss, Jena, Germany), which was adapted from the ETDRS (Early Treatment Diabetic Retinopathy Study Report Number 2) at baseline. The laser treatment involved focal treatment to all leaking microaneurysms that were located between 500 and 3000 microns from the center of the macula. The settings used for focal laser were as follows: spot diameter 50 microns, exposure time 0.1 seconds, power 50–150 mWatt. The settings were adjusted as required in order to create whitening within the wall of the microvascular lesion. Retreatment protocol was considered if DME persisted or recurred no sooner than 3 months from the time of the last treatment. No treatment was performed to the areas of retinal non-perfusion or retinal thickening. 

 Optical coherence tomography imaging was performed by a single experienced examiner with the fast macular thickness map protocol. The optical coherence tomography scan is displayed in a grid pattern that has three homocentric circles centered on the fovea. The inner circle has a radius of 1000 microns, the middle 3000 microns and the outer 6000 microns. The middle and outer circles are divided into 4 quadrants each. Therefore, the macular area is divided into 9 zones in total. The retinal thickness in each of the nine map sectors and macular volume on OCT were measured automatically using OCT software. The average inner zone thickness (IZT) and average outer zone thickness (OZT) with diameters of 3000 microns and 6000 microns were obtained by averaging the 4 inner and outer quadrants, respectively. Central subfield thickness was defined as the mean thickness in the central 1000 microns diameter according to the ETDRS layout.

 The primary outcome measures were the change in visual acuity letter score and inner zone thickness; the secondary outcome measures were the change in central subfield thickness, outer zone thickness, and macular volume at 3, 6, and 12 months. 

 Categorical variables were presented as numbers and percentages, while quantitative variables were expressed as the mean and standard deviation. The Mann-Whitney U test was used to compare differences between the two groups, and the Wilcoxon test was used to compare differences within the groups. Statistical analysis was performed using SPSS 16.0 (SPSS, Inc., Chicago, IL). The two-sided significance level was set at *p*<0.05.

## RESULTS

**Figure 1:**
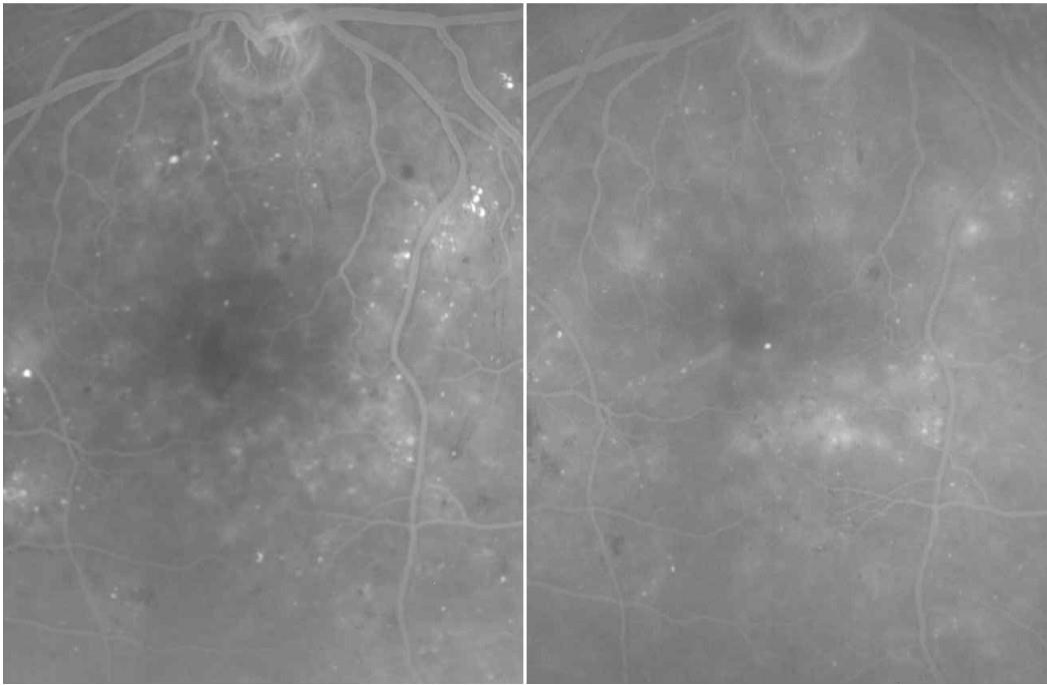
Top: Angiographic leakage prior to laser treatment. Bottom: Decrease of the angiographic leakage following laser treatment.

A total of 49 eyes of 43 patients with non-CI ME were included in this retrospective, interventional, and controlled study. Twenty-nine eyes of 25 patients with non-CI ME were treated by focal laser photocoagulation. The control group consisted of 20 eyes of 18 patients with non-CI ME. The mean number of laser treatment sessions was 1.6±0.7 (1-3) in the treatment group. Fluorescein angiography showed decreased leakage in the central macular area following a session of laser treatment (Figure 1). Baseline demographic and clinical characteristics of both groups are summarized in Table 1.

**Table 1:** Baseline demographic and clinical characteristics of the treatment and control groups.

**Variables**	**Treatment group** **(25 patients, 29 eyes)**	**Control group** **(18 patients, 20 eyes)**	***p*** ** value**
Age±SD^a^ (years)	63.2±7.9	60.4±9.1	0.28
Gender (F/M)^b^	8/17	4/14	0.48
BCVA c(ETDRS^d^ letters) (range)	74 (65-85)	76 (65-87)	0.45
IZT^e^±SD (microns) (range)	296±24 (242-346)	285±19 (248-308)	0.09
CST^f^±SD (microns) (range)	223±17 (181-248)	214±21 (173-245)	0.17
OZT^g^±SD (microns) (range)	277±25 (226-335)	265±16 (236-283)	0.11
TMV^h^±SD (mm^3^) (range)	7.9±0.6 (6.7-9.4)	7.6±0.5 (6.4-8.2)	0.07

a SD: Standard deviation;

b F/M: Female/Male;

c BCVA: Best corrected visual acuity;

d ETDRS: Early Treatment Diabetic Retinopathy Study;

e IZT: Inner zone thickness;

f CST: Central subfield thickness;

g OZT: Outer zone thickness;

h TMV: Total macular volume.

 The mean baseline VA letter score was not significantly different between the two groups (*p*=0.45). In the treatment group, the mean VA letter score did not reveal a significant change at 3, 6, and 12 months compared with the baseline (*p*=0.69, *p*=0.86, *p*=0.72, respectively). In the control group, the mean VA letter score showed a gradual decrease at 3 months and this decrease became significant at 6 and 12 months compared with the baseline (*p*=0.09, *p*=0.04, *p*=0.02, respectively). Table 2 summarizes the VA letter score changes during the follow-up period.

**Figure 2:**
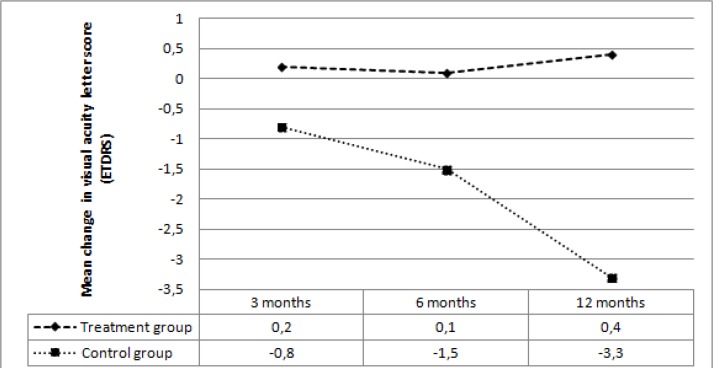
Mean change in visual acuity letter score at follow-up visits in the two groups.

 In the treatment group, the mean baseline VA letter score decreased by 0.2 at 3 months, 0.1 at 6 months, and 0.4 at 12 months. In the control group, the mean baseline VA letter score decreased by 0.8 at 3 months, 1.5 at 6 months, and 3.3 at 12 months. With regards to the mean change in VA letter score, there was no significant difference between the two groups at 3 months and 6 months (*p*=0.29, *p*=0.12, respectively); however, the difference was significant at 12 months (*p*=0.03) (Figure 2).

 At 12 months, 6 (21%) of the eyes in the treatment group and 1 (5%) eye in the control group were improved by ≥5 letters (*p*=0.12). Five (17%) of the eyes in the treatment group and 6 (30%) of the eyes in the control group were worsened by ≥5 letters (*p*=0.29). Visual acuity remained stable (+/- <5 letters) in 18 (62%) of the eyes in the treatment group, and 13 (65%) of the eyes in the control group (*p*=0.83). 

 The average baseline IZT was not significantly different between the two groups (*p*=0.09). In the treatment group, mean IZT showed a significant reduction at 3, 6, and 12 months compared with the baseline (*p*=0.001, *p*<0.001, *p*<0.001, respectively). In the control group, average IZT showed a non-significant increase at 3, 6, and 12 months compared with baseline (*p*=0.76, *p*=0.44, *p*=0.06, respectively). Table 3 summarizes the average IZT values during the follow-up times. 

**Figure 3:**
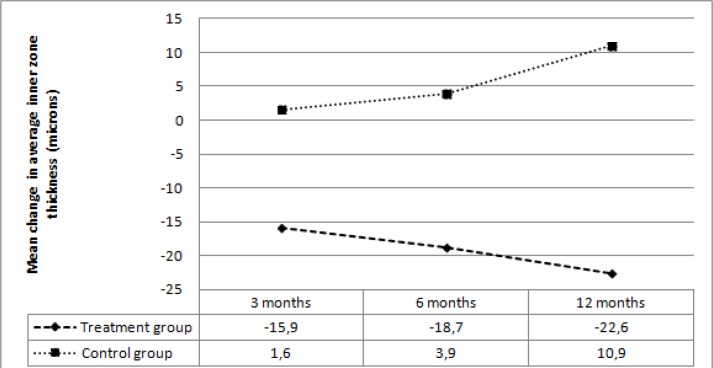
Mean change in average inner zone thickness at follow-up visits in the two groups.

 In the treatment group, average IZT decreased by 15.9 microns at 3 months, 18.7 microns at 6 months, and 22.6 microns at 12 months. In the control group, average IZT increased by 1.6 microns at 3 months, 3.9 microns at 6 months, and 10.9 microns at 12 months. The mean change in average IZT was statistically different between the two groups at 3, 6, and 12 months (*p*=0.006, *p*=0.001, *p*<0.001, respectively) (Figure 3). 

**Table 2:** Best corrected visual acuity (ETDRS letters)

**Visit**	**Treatment group**	***p*** ** value**	**Control group**	***p*** ** value**
Baseline	74 (65-85)	-	76 (65-87)	-
3 months	74 (60-85)	0.69	75 (61-85)	0.09
6 months	74 (57-85)	0.86	74 (53-84)	0.04
12 months	74 (55-85)	0.72	72 (37-84)	0.02

Data are presented as mean (min-max)

**Table 3:** Average inner zone thickness (microns)

**Visit**	**Treatment group**	***p*** ** value**	**Control group**	***p *** **value**
Baseline	296±24 (242-346)	-	285±19(248-308)	-
3 months	280±22 (234-348)	0.001	287±24 (240-328)	0.76
6 months	278±21 (240-321)	<0.001	290±27 (251-336)	0.44
12 months	273±20 (236-291)	<0.001	296±26 (252-345)	0.06

Data are presented as mean±SD (min-max)

**Figure 4:**
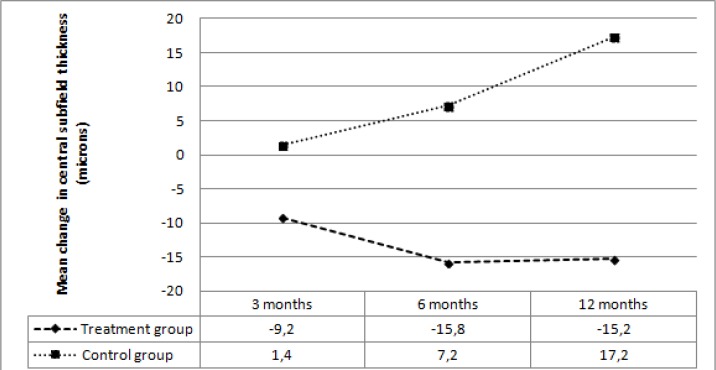
Mean change in central subfield thickness at follow-up visits in the two groups.

 The mean baseline CST, the baseline average OZT, and the mean baseline TMV were not significantly different between the two groups (*p*=0.17, *p*=0.11, *p*=0.07, respectively). During the follow-up period, a progressive reduction in mean CST, average OZT, and mean TMV was observed in the treatment group. In the control group, mean CST, average OZT, and mean TMV showed a small thickening at 3 months, with a gradual increase at 6 months and 12 months. Mean CST during the follow-up period is shown in Table 4.In the treatment group, mean CST decreased by 9.2 microns at 3 months, 15.8 microns at 6 months, and 15.2 microns at 12 months; while mean CST thickened by 1.4 microns at 3 months, 7.2 microns at 6 months, and 17.2 microns at 12 months in the control group. The mean change in CST was significantly different between the two groups at 3, 6, and 12 months (*p*=0.03, *p*=0.01, *p*<0.001, respectively) (Figure 4).

**Figure 5:**
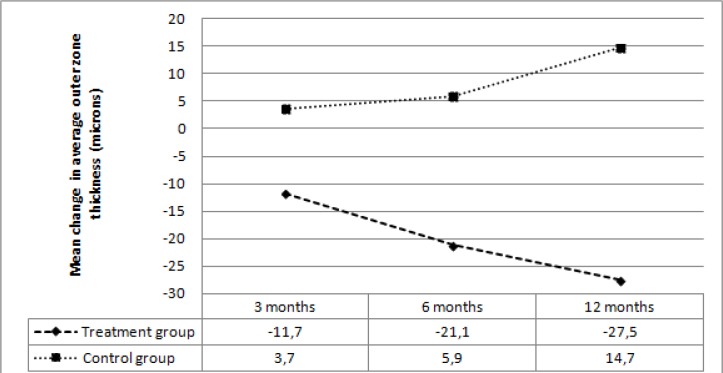
Mean change in average outer zone thickness at follow-up visits in the two groups.

**Figure 6:**
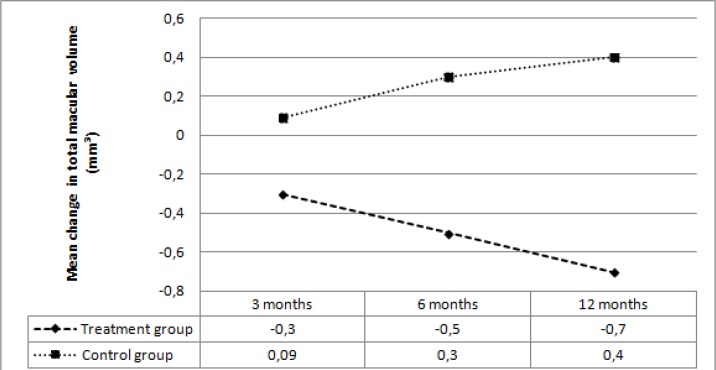
Mean change in total macular volume at follow-up visits in the two groups

 In the treatment group, average OZT decreased by 11.7 microns at 3 months, 21.1 microns at 6 months, and 27.5 microns at 12 months. In the control group average OZT increased by 3.7 microns at 3 months, 5.9 microns at 6 months, and 14.7 microns at 12 months. The mean change in average OZT was significantly different between the two groups at 3, 6, and 12 months (*p*=0.001, *p*=0.001, *p*<0.001, respectively) (Figure 5). In the treatment group, mean TMV decreased by 0.3 mm3 at 3 months, 0.5 mm3 at 6 months, and 0.7 mm3 at 12 months. In the control group, mean TMV increased by 0.09 mm3 at 3 months, 0.3 mm3 at 6 months, and 0.4 mm3 at 12 months. The mean change in TMV was significantly different between the two groups at 3, 6, and 12 months (*p*=0.004 *p*<0.001, *p*<0.001, respectively) (Figure 6). 

**Table 4:** Central subfield thickness (microns)

**Visit**	**Treatment group**	***p*** ** value**	**Control group**	***p*** ** value**
Baseline	223±17 (181-248)	-	214±21 (173-245)	-
3 months	214±25 (170-256)	0.06	215±18 (184-267)	0.53
6 months	207±31 (163-265)	0.01	221±34 (185-279)	0.21
12 months	208±27 (167-251)	0.009	236±29 (191-319)	0.01

Data are presented as mean±SD (min-max)

Table 5 and 6 summarize the mean changes in average OZT and TMV for both groups, respectively.

**Table 5:** Average outer zone thickness (microns)

**Visit**	**Treatment group**	***p*** ** value**	**Control group**	***p*** ** value**
Baseline	277±25 (226-335)	-	265±16 (236-283)	-
3 months	265±17 (224-296)	0.004	269±22 (242-287)	0.76
6 months	256±18 (220-291)	<0.001	271±20 (255-317)	0.09
12 months	249±18 (216-284)	<0.001	280±29 (246-324)	0.008

Data are presented as mean±SD (min-max)

**Table 6:** Total macular volume (mm^3^)

**Visit**	**Treatment group**	***p*** ** value**	**Control group**	***p *** **value**
Baseline	7.9±0.6 (6.7-9.4)	-	7.6±0.5 (6.4-8.2)	-
3 months	7.6±0.4 (6.6-8.7)	0.001	7.7±0.4 (6.6-8.4)	0.39
6 months	7.3±0.4 (6.4-8.1)	<0.001	7.9±0.7 (6.8-8.7)	0.06
12 months	7.2±0.5 (6.2-8)	<0.001	8.1±0.6 (6.5-8.9)	0.02

Data are presented as mean±SD (min-max)

## DISCUSSION

Clinical significant ME has been described by ETDRS studies (2,9) and divided into three groups: type 1, type 2 and type 3. Type 1 includes the center-involving ME, whereas type 2 and type 3 represent the non-CI ME types. The progression of non-CI ME has been reported as an increase in central macular thickness at least 50 microns from baseline as found in 38% of untreated eyes in two years. It has therefore been suggested that non-CI ME probably represents a precursor stage of center-involved clinically significant ME (10). Current ETDRS guidelines still remain appropriate and there are no new recommendations for the treatment of non-CI ME to date (5). Modified ETDRS (mETDRS) focal/grid laser photocoagulation protocol in center-involving ME, adopted from the original ETDRS, is the widely used technique by most retina specialists. Modified ETDRS treatment is based on treating areas of thickened macula and areas of non-perfusion and leaking microaneurysm with less intense and smaller burns than in the original ETDRS treatment. A prior DRCR.net study evaluating macular photocoagulation regimens demonstrated that the mETDRS laser approach was more effective in reducing retinal thickening at 12 months than a mild macular grid laser technique in which small mild burns were placed throughout the macula. In the same study, 25% of the patients treated with the mETDRS technique gained 15 or more letters, and only 6% of patients treated lost 15 or more letters (11). 

 Despite the decrease in extent of the leakage following focal/grid photocoagulation, undesirable events such as central scotomata and loss of central vision may occur and are mostly caused by the progressive enlargement of the laser scars (12). Recently, it has been proposed that a useful therapeutic response is provided by the viable RPE cells surrounding the burned areas, not by laser-killed RPE cells in the response to thermal injury (13). 

 The aim of DME treatment should primarily be improvement or stabilization of VA and secondarily prevention of further vision loss. Therefore, new laser treatment strategies should be developed to minimize chorioretinal damage in eyes with less severe retinal thickening at the center of the macula, while maintaining similar treatment efficacy. The first study examining the impact of focal/grid laser photocoagulation consisted of a combination of focal treatment to individual-leaking microaneurysms and grid treatment to areas of diffuse leakage and capillary non-perfusion in non-CI ME was released by the EDTRS group (14). They found that focal/grid laser photocoagulation tended to reduce the percentage of patients that had either moderate visual loss or had visual acuity worse than 20/100 after 5 years of laser treatment. The second study by Scott *et al*., (15) showed that one year after the focal/grid laser treatment, the mean VA remained unchanged, while the mean CST was reduced by 10 microns. In these studies, changes in retinal thickness have been assessed by colored stereoscopic fundus photographs, OCT, and FA. In clinical practice, decisions on treatment options are most likely to be based on the combination of OCT and VA (4). Fluorescein angiography assessment may be required in specific situations such as development of unexplained visual loss. Therefore, we followed the patients by VA measurements and retinal thickness changes measured with OCT while evaluating their response to therapy. 

 In agreement with the previously mentioned studies, we found that focal laser photocoagulation had a stabilizing effect on VA. Final visual outcomes in our study showed that 62% of eyes maintained their baseline VA and 21% of eyes showed an improvement in VA in the treatment group. In the control group, VA stabilized in 65% eyes and improved in 5% of eyes at 12 months. This accounts for an overall positive effect on VA in 83% of the treated eyes versus 70% of eyes without treatment. A reduction in CST by 15 microns in the treated eyes at 12 months suggested that there was some center-involved edema at baseline. We found beneficial effect in reducing the retinal thickening in the inner zone and outer zone, and also in volume measurements. Conversely, we observed that the retinal thickness measurements and TMV in the control group showed a small gradual increase during the study period.

 The precision of the results of the present study is limited by its relatively small study population and retrospective nature. Prospectively designed studies with long-term follow-up are needed to provide additional data.

 In conclusion, we assessed a less aggressive laser therapeutic strategy that was limited to photocoagulation of microaneurysms for non-CI ME in this study. Taken together, we believe that focal laser treatment applied directly to angiographically leaking microaneurysms has a benefit of stabilizing the visual acuity and reducing the retinal thickening at 12 months compared to observation alone. We suggest an early intervention with focal laser treatment in eyes with non-CI ME.
